# A randomized controlled double blind trial comparing the effects of the prophylactic antibiotic, Cefazolin, administered at caesarean delivery at two different timings (before skin incision and after cord clamping) on both the mother and newborn

**DOI:** 10.1186/s12884-017-1526-y

**Published:** 2017-10-03

**Authors:** Chinta Annie Jyothirmayi, Ajay Halder, Bijesh Yadav, Santosh Thomas Samuel, Anil Kuruvilla, Ruby Jose

**Affiliations:** 1 Department of Neonatology, Christian Medical College, Vellore, India; 2 Department of Obstetrics and Gynecology Unit IV, Christian Medical College, Vellore, India; 3 Department of Biostatistics, Christian Medical College, Vellore, India; 4 MLL Hospital, Madanapalle, Chittoor, Andhrapradesh India

**Keywords:** Prophylactic antibiotic, Caesarian delivery, Pre-incision, Post-incision

## Abstract

**Background:**

Caesarean delivery (CD) increases the risk of postpartum infection by 5 to 20 fold. Prevention of surgical site infection (SSI) is the goal of antibiotic prophylaxis. This study was carried out to assess the optimum timing for prophylactic antibiotic administration and to assess the amount of the antibiotic crossing the placental barrier.

**Methods:**

Eligible mothers were recruited, after informed consent, once the decision for CD was made. Each mother received two injections, one prior to skin incision and one after cord clamping, (one being the study drug Cefazolin, and the other, a placebo) based on the randomization code. Demographic, maternal and neonatal monitoring data until discharge from hospital, and at the 6 weeks postpartum visit were collected. Levels of the prophylactic antibiotic were measured from the cord blood in every 8th neonate. The objective of the study was to compare the effects of the prophylactic antibiotic, intravenous Cefazolin 1 g, administered at Caesarean delivery (CD) at two different timings (before skin incision and after cord clamping) on both the mother and newborn. The secondary outcomes that were followed up were the number of maternal and neonatal readmissions. An appropriate test for significance, Fisher’s exact test was used to find the association between risk variables and outcome.

**Results:**

The total numbers of mothers enrolled were 1106, of whom 553 mothers received antibiotic prior to skin incision (pre-incision) and 543 mothers received antibiotic after cord clamping (post-incision). The pre-incision group had significantly less febrile illness (RR = 0.48, 95% CI: 0.29 - 0.80) and SSI (RR = 0.14, 95% CI: 0.04 - 0.53) when compared with the post- incision group. The post-incision group significantly had >7 days hospital stay when compared to the 4-7 days stay of the pre-incision group (*p* = 0.005).There were no differences in any of the neonatal outcomes. The quantity of the antibiotic in the cord blood was only 2-3%.

**Conclusions:**

Pre incision prophylactic antibiotic protected the mother from SSI and febrile illness and decreased the hospital stay significantly.

**Trial registration:**

The Clinical Trials Registry India (CTRI) was [CTRI/2016/03/006710 dated, 04/03/2016].

## Background

Caesarean delivery (CD) is considered an important risk factor for postpartum infection. When compared to women who deliver vaginally, those delivered by (CD) face a 5 to 20 fold increase in risk of infection [1]. Prevention of surgical site infection (SSI) by decreasing the burden of microorganisms at the surgical site during the operative procedure is the goal of antibiotic prophylaxis [1]. The efficacy of antibiotic prophylaxis for reducing SSI is well known [2].

Prophylactic antibiotics are expected to work in conjunction with the antiseptic measures taken before and during surgery [3, 4]. It is suggested that antibiotic given 30 min to 1 h before skin incision will cause adequate bactericidal concentration in the system before inoculation. Prophylactic antibiotics act mainly by destroying the bacteria and slowing production of bacterial proteases, thus preventing the attachment of bacteria to the mucosal surfaces. The greatest therapeutic effect occurs when antibiotics are administered just before or coincident when maximal bacterial contamination and tissue trauma occurs.

At CD, in our Institution, the prophylactic antibiotic was being administered after cord clamping, so that it did not reach the foetal circulation. Concerns of masking signs of sepsis in babies, developing resistance to antibiotics and masking organisms in blood culture because of the transplacental transfer of the drug was the main reason for administration of the drug after cord clamping. However, recent studies suggested that, giving the drug prior to skin incision would significantly decrease the incidence of maternal infection without causing harm to the baby [5, 6]. Administration of IV antibiotics prior to skin incision definitely showed a decrease of 9% in the rate of maternal infections with no increase in the rate of neonatal sepsis, duration of hospital stay for the infant or any complications secondary to the administration of antibiotic to the mother [7].The objectives of the study were:

1. To compare the effects of the prophylactic antibiotic, intravenous Cefazolin1gm, administered at Caesarean delivery (CD) at two different timings(30 min to 1 h) before skin incision and immediately after cord clamping) on both the mother and newborn.
2. To quantify the amount of antibiotic that has crossed the placental barrier in to the cord blood.

## Methods

This randomized controlled double blinded trial was carried out at the Christian Medical College and Hospital (CMCH), Vellore in the Departments of Obstetrics and Gynaecology and Neonatology, over a period of 7 months from March 2013 to September 2013. CMCH is a tertiary care centre situated in Vellore, in South India, and has around 15,000 deliveries a year, catering mostly to women from the surrounding 2 states of Tamil Nadu and Andhra Pradesh.

All mothers more than or equal to 37 completed weeks of gestational age who had a decision made for CD (elective and emergency)were included in the study, after informed consent from each participant. The study was cleared by the Institutional Review Board and the Ethics Committee of Christian Medical College, Vellore. [IRB Min no. 8084 dated 21.11.2012]. The Clinical Trials Registry India (CTRI) was [CTRI/2016/03/006710 dated, 04/03/2016].

Mothers with known allergy to Cephalosporin or Penicillin, those who received antibiotics within a week prior to CD, those with altered liver or renal functions and those on antiepileptic medication were excluded from the study. Babies with known major congenital anomalies were not included as well. Women undergoing CD were randomized to receive both single dose of IV Cefazolin 1 g and placebo. As per the randomization, every mother was given IV Cefazolin1gm either 30 min to 1 h, prior to skin incision, with placebo administered within a minute after cord clamping or placebo administered 30 min to 1 h prior to skin incision and Cefazolin 1 g IV, within a minute after cord clamping. The randomization covers with the blinded drugs were opened in the Operation Theatre (OT).The medications were administered by the Anaesthetist appropriately as per the randomisation. Cord blood was collected in every 8th neonate to estimate the amount of antibiotic levels in the neonate. The cord blood (5 ml) was collected soon after delivery of the baby in a blood collecting test tube, centrifuged and the serum was refrigerated for analysis. The drug level (Cefazolin) was analysed by a developed and validated High Pressure Liquid Chromatography (HPLC) with Ultra violet protection method in the clinical pharmacological unit. Thus, the investigator, the patient and the Labour Room personnel were blinded to the drug used. After CD, all the outcomes listed below were assessed until discharge from hospital and at the first postpartum visit at 6 weeks and noted. In addition, basic demographic and obstetric data were noted as well. The primary maternal outcomes assessed were the presence of SSI, endometritis, UTI and length of hospital stay. The primary outcomes assessed for the neonate were incidence of early and late onset sepsis, oral thrush, increased frequency of stools, necrotizing enterocolitis (NEC) and length of hospital stay. The secondary outcomes in the mother and the neonate that were followed up were the number of readmissions and maternal / neonatal death due to sepsis until 6 weeks postpartum.

The sample size to compare the effect of IV Cefazolin on the mother and infant on infections was found to be 600 in each arm with 90% power, at 5% level of significance, an anticipated difference of 9% in each group and with a 20% loss to follow-up.

Permutated Block Randomization of sizes 2, 4 and 6 was done for treatment allocation using SAS 9.1.3. Pre numbered or coded identical containers for the antibiotics and the placebo prepared by pharmacy which were administered serially to patients. Randomization was done according to computer generated blockrandomization.1200 mothers planned for CD were recruited into the study, but only 1106 of those could be analysed. Five hundred fifty three mothers received the antibiotic prior to skin incision and placebo after cord clamping referred to as Pre-incision group in the study. The remaining 543 received placebo prior to skin incision and IV Cefazolin after cord clamping referred to as Post-incision in the study.

### Operational definitions

#### Surgical site infections (SSI)

Evidence of clinical signs and symptoms of infection with or without microbiological evidence, based on the CDC definition that describes three levels of SSI [8].

#### Endometritis

Inflammation of the inner layer of the uterus, diagnosed clinically by the presence of pyrexia, uterine tenderness, sub involution of the uterus and foul smelling lochia along with or without microbiological evidence.

#### Urinary tract infections (UTI)

Presence of clinical symptoms of dysuria, increased frequency of micturition and pyrexia along with or without microbiological evidence.

#### Early on set neonatal sepsis

Presence of any or a combination of features of sepsis namely hypo or hyperthermia, poor feeding, lethargy, dusky extremities, tachypnea or apnea within the first 72 h of life.

#### Late onset neonatal sepsis

Presence of the above features after the first 72 h of life until the first 1 week of life.

#### Oral thrush

Presence of white plaque like rash on buccal mucosa caused mainly by fungal organism giving rise to difficulty in feeding.

#### Necrotizing enterocolitis (NEC)

Necrosis of the gut seen as temperature instability, poor feeding, lethargy, .vomiting, abdominal distension, feed intolerance, sometimes with bloody stools.

#### Length of hospital stay

Defined as the number of days, the day mother is admitted into the hospital and for the neonate, the day neonate is born, until mother/neonate is discharged from hospital.

#### Maternal and neonatal readmission

Any admission to the same hospital or any other facility after first discharge from hospital.

#### Neonatal deaths due to sepsis

Death following sepsis which has been confirmed by positive growth on blood culture/high C reactive protein levels with leukocytosis or leucopoenia or thrombocytopenia.

All maternal outcomes were assessed by the Consultant Obstetrician who was assigned to the post- operative ward, who had a minimum of 4 years of clinical experience. All newborns were assessed by the Registrar/Neonatologist on duty, who assigned the APGAR score as well. If the Neonatologist or Registrar was not available on site in an emergency, the Labour room Registrar assessed the APGAR score. Sepsis in the neonate was assessed by a Consultant Neonatologist with a minimum of 4 years of clinical experience.

All monitoring details were collected by the Principal Investigator.

### Statistical methods

Data entry was done into EpiData software in the Neonatal department. Analysis was done using SPSS 16 software. All categorical variables were summarized using frequencies and percentages and continuous variables were using mean and standard deviation. The association between risk variables and the primary outcome (Pre-incision and Post-incision) were tested using Fisher’s exact test. Adverse effects with IV Cefazolin, though thought to be unlikely were monitored and all data were submitted to the institutional Data Safety Monitoring Board for review at the end of the trial.

## Results

**Fig. 1:**
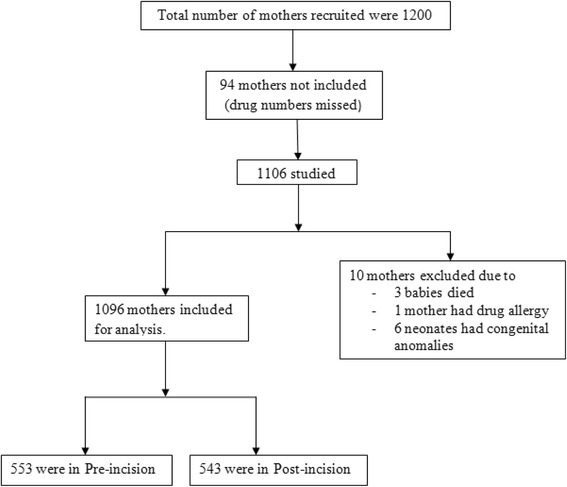
The scheme of patient recruitment

One thousand two hundred mothers were recruited but details of only 1106 mothers could be used for analysis over the study period of 7 months. Ninety four mothers could not be included after randomization, as the drug numbers were inadvertently missed. Among 1106 mothers recruited for the study, 553 mothers received IV Cefazolin prior to skin incision and 543 mothers received antibiotics after cord clamping. Ten mothers were excluded at the end of the study for the following reasons; 1 mother developed allergy to IV Cefazolin, 3 neonates died (not due to sepsis) and 6 neonates had congenital anomalies (undiagnosed antenatally). The scheme of patient recruitment presented in Fig. 1.

### Demographic data of the mothers and neonates

The demographic data for mothers and neonates are presented in Table 1. There were no differences in demographic characteristics of the mothers and neonates across the two groups. However, on the whole there were more mothers who underwent emergency caesarean delivery (75.7%) than elective (24.3%).

**Table 1:** Demographic Variables of Mothers and Neonates

Variables	Pre incision		Post incision		*P* value
	n	%	n	%	
Parity:					
Primiparous	252	45.6	224	41.3	0.15
Multiparous	301	54.4	319	58.7	
Risk Factors:					
Hypertension	98	17.7	96	17.7	
Diabetes	86	15.6	71	13.1	0.63
Hypothyroidism	9	1.6	7	1.3	
None	360	65.1	369	68.0	
Type of CD:					
Elective	131	23.7	136	25.0	0.60
Emergency	422	76.3	407	75.0	
Indication for CD:					
Fetal distress	165	29.8	181	33.3	
Abnormal lie	56	10.1	50	9.2	
Cord Prolapse	1	0.2	1	0.2	0.41
Previous Caesarean	179	32.4	190	35.0	
Arrest of dilatation	55	9.9	52	9.6	
Failed Induction	81	14.6	57	10.5	
Scar rupture	16	2.9	12	2.2	
Sex of baby:					
Male	279	50.5	278	51.2	0.80
Female	274	49.5	265	48.8	
APGAR at 5 min:					
Normal	535	96.7	522	96.1	0.58
Abnormal	18	3.3	21	3.9	
Feeds in hours:					
< 12	524	94.9	527	97.1	
12-24	23	4.2	10	1.8	0.08
> 24	5	0.9	6	1.1	

The mean (SD) gestational age of the mothers at delivery was 38.5(1.8). Of the 1096 mothers, 479 (43.5%) were primiparas and 622 (56.5%) were multiparas. Emergency CDs were done on 834 (75.7%) and the rest were elective CDs 267 (24.3%). Hypertensive disease complicated 194 (17.6%), Diabetes was present in 159 (14.4%), and hypothyroidism in 16 (1.5%). The indications for CD was mostly for previous CD in 371(33.7%), non reassuring fetal heart status in 348 (31.6%), labour dysfunction (arrest of dilatation, failed Induction) in 108 (9.8%), transverse or oblique lie of the fetus in 106 (9.8%), scar rupture in 28 (2.5%) and cord prolapse in 2(0.2%).

There were 553 neonates in the Pre-incision group and 543 in the Post-incision group, of whom 557 were male and 539 were female babies. The mean (SD) birth weight of the babies was 3 kg (0.65). There were 12 (2.2%) and 7 (1.3%) of neonates weighing 1-2 kg in Pre-incision and Post-incision respectively. Babies weighing more than 4 kg were less than 2% in both groups.

The mean (SD) APGAR scores of the babies at birth were 8 (1).Most of the newborns, 524 (94.9%) in the Pre-incision group and 527 (97.1%) in the Post-incision group had started feeds within 12 h after birth. Some of the newborns, 23 (4.2%) in the Pre-incision and 10 (1.8%) in Post-incision groups started feeds in 12 to 24 h. The reasons for the delay in.

commencing feeds were, due to transfer of the baby to the nursery either for tachypnea, grunting or hypoglycaemia requiring IV fluids.

### Results of the primary maternal outcomes

The outcome data for mothers and neonates are presented in Table 2.The primary maternal outcomes that were assessed were, the presence of post operative infectious morbidity comprising of SSI, endometritis, UTI and length of hospital stay. When considering the primary maternal outcomes, the mothers who received prophylactic antibiotics pre incision, had less post operative complications such as febrile illness and SSI.

**Table 2:** Outcome Variables of Mothers and Neonates

Variables	Pre incision		Post incision		RR	95% CI	P value
	n	%	n	%			
*Mothers*							
Hospital days:							
1-3	2	0.4	5	0.9	0.56	0.17 – 1.80	0.28
4-7	539	97.6	510	93.9	1.00		
> 7	11	2.0	28	5.2	0.55	0.33 – 0.91	0.005
Gestational Age:							
< =37	136	25.0	126	23.2	1.05	0.92 – 1.20	0.48
> 37	407	75.0	418	76.8	1.00		
Blood culture:							
Yes	23	4.2	70	12.9	0.47	0.33 – 0.67	<0.001
No	529	95.8	473	87.1	1.00		
Proven sepsis:							
Yes	8	1.4	8	1.4	0.99	0.60 – 1.62	0.97
No	544	98.6	535	98.5	1.00		
Post op Complications;							
Febrile illness	11	2.0	32	5.9	0.48	0.29 – 0.80	0.005
SSI	2	0.4	25	4.6	0.14	0.04 – 0.53	<0.001
Endometritis	5	0.9	14	2.6	0.49	0.23 – 1.05	0.02
UTI	16	2.9	21	3.9	0.81	0.56 – 1.18	0.24
None	518	93.8	451	83.1	1.00		
Readmission:							
Yes	7	1.3	11	2.0	0.77	0.43 – 1.39	0.35
No	543	98.2	532	98.0	1.00		
*Neonates*							
NICU Admission:							
Yes	23	4.2	17	3.1	1.15	0.87 – 1.50	0.42
No	530	95.8	526	96.9	1.00		
Hospital days:							
1-7	528	95.7	508	93.6	1.00		
> 7	24	4.3	35	6.4	0.80	0.58 – 1.09	0.14
Weight of Baby (Kg):							
1-2	12	2.2	7	1.3	1.23	0.86 – 1.75	0.36
2-3	271	49.0	257	47.3	1.00		
3-4	246	47.7	269	49.5	0.93	0.82 – 1.05	0.26
> 4	6	1.1	10	1.8	0.73	0.39 – 1.38	0.32
Blood culture:							
Yes	58	10.5	66	12.2	0.91	0.75 – 1.12	0.39
No	495	89.5	477	87.8	1.00		
*Neonates*							
Proven Sepsis:							
Yes	5	0.95	5	0.95	0.99	0.53 – 1.85	0.99
No	547	99.1	538	99.1	1.00		
Weight gain at review:							
Yes	540	97.8	526	96.9	1.22	0.79 – 1.90	0.35
No	12	2.2	17	3.1	1.00		
Readmission:							
No	533	96.9	517	95.2	1.28	0.83 – 1.87	0.16
Yes	17	3.1	26	4.8	1.00		

*RR* Relative Risk

The pre-incision group had significantly less febrile illness (RR = 0.48, 95% CI: 0.29 – 0.80) and SSI (RR = 0.14, 95% CI: 0.04 – 0.53) compared with the post incision group. The total number of UTI’s was 16 (2.9%) in the Pre-incision group and 21 (3.9%) in the Post incision group which was not significant. Hospital stay of more than 7 days which indirectly revealed that delay was probably due to some infectious morbidity was 11 (2%) in the Pre-incision versus 28 (5.2%) in the Post-incision group. The post-incision group compared to the pre-incision group had significant more mothers with a length of stay >7 compared to having a length of stay of 4-7 days (RR = 0.55, 95% CI: 0.33 – 0.91, *p* = 0.005). The pre incision group had a lower risk of a positive growth of significant micro organisms in blood culture than the post incision group (RR = 0.47 95% CI: 0.33 – 0.67).Blood culture reported significant growth in 23 (4.2%) mothers in the Pre-incision and in 70 (12.9%) mothers in the Post-incision group, signifying increased rate of infections in the post incision group. Readmission rates after discharge from hospital were similar in both groups.

### Results of the primary outcomes in the neonate

The primary outcomes assessed for the neonate were incidence of early and late onset sepsis, oral thrush, increased frequency of stools, and length of hospital stay. There were no neonates with oral thrush or NEC in either group. Admissions of the neonates to the NICU were for various reasons, for example, low APGAR at birth, transient tachypnea of newborn, hypoglycaemia, meconium aspiration and dysmorphism. The numbers of admissions to NICU were more from the Pre-incision group, 23 (4.2%) as compared to the Post-incision group 17 (3.1%), but this was not statistically significant (*p* = 0.42). Most of the neonates 528 (95.7%) and 508 (93.6%) stayed in hospital 4 to 7 days in the Pre-incision and Post-incision groups respectively. Blood culture was found positive in 58 (10.5%) in the Pre-incision and 66 (12.2%) in the Post-incision group which was not statistically significant (*P* = 0.39). Proven sepsis by other tests was present in 5 (0.95%) in both groups. None of the babies had any other immediate complications.

### Results of the secondary outcomes in the mother and newborn

There were no maternal deaths or readmissions after discharge and the percentage of infants who had weight gain at 6 weeks was similar. As for the newborns, the readmission rates were similar 17 (3.1%) and 26 (4.8%) in both groups respectively and weight gain at 6 weeks were similar. There were no neonatal deaths due to sepsis at the postpartum visit.

The cord blood level of Cefazolin in 19 of Group 1 (Pre-incision) babies had <1 mg/dl. Forty three babies had cord blood level of Cefazolin between 1 and 10 mg/dl, 60 babies had levels between 10 and 20 mg/dl, and 17 babies had levels between 20 and 30 mg/dl. The maternal blood levels however were not checked.

The toxic level of the drug in the blood is not known. However the study showed that on an average, only 2-3% of the drug administered to the mother reached the baby. This may suggest that the drug may not cause harm to the baby since very little amount reaches the baby once administered to the mother, although the study was not powered for this outcome.

## Discussion

Infectious morbidity for the mother, consisting primarily of endomyometritis and wound infection remains a leading cause of post operative complications. Infectious morbidity following CD remains among the top five causes of mortality in mothers’ worldwide, causing a huge burden to the society [9].

In the study by Sullivan et al. [7], on 357 women, decreased total infectious morbidity in the study group was found (RR = 0.4, 95% CI = 0.18 to 0.87). There was a decreased endometritis risk as well in the pre incision group (RR = 0.2, 95% CI = 0.15 to 0.94).There was no increase in neonatal sepsis (*P* = 0.99), sepsis work up (*P* = 0.96) or increased hospital stay (*P* = 0.17). In the present study, the results were almost similar in that, the incidence of SSI was decreased and duration of hospital stay was decreased with antibiotics given Pre-incision than after cord clamping. Unlike the study by Sullivan et al. [7], in the present study, the incidence of endometritis was similar in both groups. Administration of prophylactic cefazolin prior to skin incision resulted in a decrease in both endometritis and total post caesarean infectious morbidity, compared with administration at the time of cord clamping. This dosing did not result in increased neonatal septic workups or complications.

The decrease in SSI in the study by Owens et al. [10], compares well with the present study, though the incidence of endometritis does not. There was a 3.9% decrease in the incidence of wound infections and 3.6% decrease in endometritis in the group which received antibiotics prior to skin incision as compared to a 2.2% decrease in the incidence of wound infection and 2.5% decrease in the incidence of maternal endometritis in the other group and this was statistically significant (*p* = 0.03) [10].

Baasqee et al. [11], reporting on 2313 women and 2345 newborns showed that preoperative administration was associated with a significant 41% reduction in the rate of endometritis compared with intra operative administration (RR = 0.59, 95% CI = 0.37-0.94). In the preoperative group, there were non-significant reductions in the rates of wound infection (RR 0.71,95% CI = 0.44-1.14), maternal febrile morbidity (RR = 0.94, 95% CI = 0.46-1.95), neonatal sepsis (RR = 0.81, 95% CI = 0.47-1.41), neonatal septic work-up (RR = 0.93, 95% CI = 0.71-1.21) and neonatal intensive-care unit admission (RR = 0.92, 95% CI = 0.65-1.28). There were non-significant increases in the rates of maternal pyelonephritis (RR = 1.09, 95% CI = 0.49-2.43) and neonatal pneumonia (RR = 3.36, 95% CI = 0.55-20.47).

This was different from the present study, which did not reveal decrease in the incidence of endometritis.

Faribaet al. [12], in their study on 410 patients found no difference in the incidence of postpartum endometritis (RR = 0.34, 95% CI = 0.04 to 3.24), which was similar to the present study. There was no difference in the incidence of wound infection (RR = 3.06, 95% CI = 0.13 to 74.69) and total puerperal morbidity (RR = 1.02, 95% CI = 0.47 to 2.22), unlike the present study. There was no difference in the incidence of neonatal sepsis (RR = 0.34, 95% CI = 0.04 to 3.24), septic workup (RR = 0.41, 95% CI = 0.08 to 2.07), or intermediate NICU admission (RR = 0.73, 95% CI = 0.24 to 2.26), which was similar to the present study.

Small *et al.* [13], in the Cochrane database publication, concluded that endometritis was reduced by two thirds to three quarters and a decrease in wound infection was also identified. Prophylactic antibiotics given to all women undergoing elective or non-elective cesarean section is clearly beneficial for women but there is uncertainty about the consequences for the baby. In the present study, infectious morbidity was definitely decreased and no adverse effect was found for the neonate.

The limitations of the study were that, though the sample size calculated was 1200 mothers, the actual numbers fell short. The study was powered only for the main maternal outcomes.

The operational definitions for some of the maternal and neonatal outcomes were based on clinical signs and symptoms, and not on any laboratory tests.

## Conclusion

Intravenous antibiotics can be administered to mothers prior to skin incision for Caesarean section and this decreases the incidence of the overall postoperative infectious morbidity in the mother. No significant adverse effects were seen in neonates when intravenous Cefazolin was administered prior to skin incision, although the present study was not powered for neonatal morbidity.

## Abbreviations

CD: Cesarean delivery
CMCH: Christian Medical College and Hospital
CTRI: The Clinical Trials Registry India
IRB: Institutional Review Board
NEC: Necrotizing enterocolitis
NICU: Neonatal intensive care unit
OT: Operation theatre
RR: Relative Risk
SSI: Surgical site infection
UTI: Urinary tract infection
